# Knowledge, attitudes and misconceptions of Italian healthcare professionals regarding fever management in children

**DOI:** 10.1186/s12887-018-1173-0

**Published:** 2018-06-18

**Authors:** Elena Chiappini, Antonina Marta Cangelosi, Paolo Becherucci, Monica Pierattelli, Luisa Galli, Maurizio de Martino

**Affiliations:** 1 Department of Health Sciences, Anna Meyer Children’s University Hospital, University of Florence, Viale Pieraccini, 24, 50100 Florence, Italy; 2 Primary Care Paediatrician, Florence, Italy

**Keywords:** Fever, Children, Antipyretics, Thermometer, Paracetamol, Ibuprofen

## Abstract

**Background:**

Fever phobia is still a major issue in paediatrics. We report knowledge of a sample of Italian paediatricians performed six years after the release of the Italian guidelines for the management of fever in children (IFG).

**Methods:**

A questionnaire, developed following the IFG recommendations and previously administered to 300 paediatricians in 2012, was proposed to all the paediatricians attending the 2015 National Congress of Practice Paediatrics, held in Florence, Italy. Changes in answers over time were analyzed.

**Results:**

70.2% (562/800) paediatricians returned the questionnaire. The recommended site and device for body temperature measurement in children > 1 year was correctly chosen by 89.3% of participants (vs. 80.7% of 2012 participants; *P* < 0.001), but with children aged less than 1 year the correct answer was selected only by the 50.3% (vs. 39.3% of 2012 participants: *P* < 0.001).

Use of physical methods was still incorrectly recommended by 51.6% of paediatricians (vs. 63.6% in 2012; *P* < 0.001). Use of antipyretics according to discomfort was adopted only by 38.2% of participants, while 12.2% of them recommended alternate use of antipyretics. These proportions were substantially stable since 2012 (45 and 11% respectively), rectal administration of antipyretics only in case of vomiting was correctly recommended by 86.8% of paediatricians vs. 74.7% in 2012 (*P* < 0.001).

**Conclusion:**

Improvements in some pediatricians’ misconceptions were observed over time. However, some incorrect habits persist. Further studies are needed to better understand the “weak points” of the communication between Scientific Societies and paediatricians in order to impact everyday clinical practice.

## Background

Since the 1980’s, when Barton Schmitt coined the term “fever phobia” [1], several studies have been published in this regard, reporting its presence both among healthcare professionals and parents/tutors, in Western countries as well as in limited resource settings [2, 3]. Despite the dissemination of several international guidelines [4, 5], poor knowledge about the correct use of antipyretics still persists among paediatricians. The results of the most recent surveys performed in Italy and in other European countries show improvements, but also the potential for further optimization [2, 6]. The present study investigated changes in knowledge/misconceptions over time by surveying a large sample of paediatricians, comparing results with those obtained in a previous similar national survey [6].

## Methods

### Study design

A survey was conducted including paediatricians attending the National Congress of Practice Paediatrics, held in Florence in November 2015. All the paediatricians attending the conference received an anonymous, standardized and self-administered paper-based questionnaire. They were requested to participate to the survey by returning the filled questionnaire to the conference registration desk. Results were entered into an electronic database, analyzed and compared with those obtained from a previous similar survey, performed in 2012, at the 12th National Congress of the Italian Society of Paediatric Infectious Diseases and based on the same questionnaire (Appendix 1) [6].

### Questionnaire

The questionnaire was developed on the basis of previous similar surveys [6, 7], and in consideration of the IFG recommendations [4]. The questionnaire consisted in multiple choice questions, as previously described [6]. Briefly, the main topics included: methods and devices recommended for the measurement of body temperature, knowledge regarding the use of physical methods and antipyretics.

#### Statistical analysis

The results were expressed as absolute numbers, percentages and 95% confidence intervals (95% CIs) were calculated. The χ^2^ or the Fischer’s exact test (2 grades of freedom) were used in order to calculate differences among responses between the years 2012 and 2015. SPSS software package was used and *p* value < 0.05 was considered as statistically significant.

The study was not commercially sponsored.

## Results

The questionnaire was returned by 562/800 (70.%) of participants; the majority (91.9%; 517/562) were primary care paediatricians; 16 (2.8%) were hospital paediatricians, and 29 (5.1%) residents/other; 393 (69.9%) participants declared to be aware of IFG.

### Methods for body temperature measurement

In children under one year of age, axillary site was correctly chosen by an increased number of paediatricians over time: 50.3% in 2015 vs. 39.3% in 2012 (*p* < 0.0001). However, the rectal measurement, which was discouraged by the IFG because considered invasive, was still commonly adopted: the proportion of participants who recommended rectal measurement in children under one year of age was 41.3% in 2012 and 34.2% in 2015 (*p* = 0.002) (Table 1).

**Table 1:** Temperature monitoring site/method used by paediatricians participating in the 2012 and 2015 surveys and type of thermometer recommended

	2012 n (%; 95% CI) *n* = 300	2015 n (%; 95% CI) *n* = 562	*P*
*Children < 1 year of age*			
Axillary*	118 (39.3; 33.8–44.9)	283 (50.4; 46.2–54.5)	0.001
Rectal	124 (41.3; 35.8–46.9)	192 (34.2; 30.2–38.1)	0.020
Groin crease	38 (12.7; 8.9–16.4)	71 (12.6; 9.9–15.4)	0.980
Oral	0 (0.0; 0.0–0.0)	1 (0.2; 0.0–0.5)	0.950
Auricular	18 (6.1; 3.3–8.7)	7 (1.2; 0.3–2.2)	< 0.0001
Forehead	2 (0.6; 0.0–1.6)	8 (1.4; 0.4–2.4)	< 0.0001
*Children > 1 year of age*			
Axillary*	242 (80.7; 76.2–85.1)	502 (89.3; 86.8–91.9)	0.0003
Rectal	9 (3.0; 1.1–4.9)	20 (3.6; 2.0–5.1)	0.660
Groin crease	15 (5.0; 2.5–7.5)	24 (4.3; 2.6–5.9)	0.630
Oral	2 (0.7; 0.0–1.6)	1 (0.2; 0.0–0.5)	0.240
Auricular	29 (9.6; 6.3–13.0)	6 (1.1; 0.2–1.9)	< 0.0001
Forehead	3 (1.0; 0.0–2.1)	10 (1.8; 0.7–2.9)	0.370
*Type of recommended thermometer*			
Digital*	203 (67.7; 62.4–73.0)	385 (68.5; 64.7–72.3)	0.430
Auricular	15 (5.0; 2.5–7.5)	9 (1.6; 0.6–2.6)	0.003
Other**	32 (10.6; 7.2–14.2)	168 (29.8; 26.1–33.7)	< 0.0001

Note: * right answer according to the Guidelines of the Italian Paediatric Society

**mercury, skin infrared, plastic streap placed forehead, dummy-pacifier style thermometers

In children > 1 year of age, a correct answer indicating axilla as the preferred site for temperature measurement was provided by 89.3% of paediatricians vs 80.7% in the 2012 survey (*p* = 0.0003).

Considering the type of thermometer recommended, the digital one was the most widely recommended (68.5%); while the use of auricular thermometer decreased overtime and was only 1.6% in 2015 vs 5.0% in 2012 (*P* = 0.003) (Table 1).

### Use of physical methods and antipyretics

Wet bandages, ice bags and other physical methods (discouraged by IFG) were never recommended by 48.0% of paediatricians in 2015, with a significant increase from the results reported in the 2012 survey (36.4%; *P* < 0.0001) (Table 2).

**Table 2:** Use of physical methods and antipyretics among paediatricians participating in the 2012 and 2015 surveys

First choice drug			
	2012 n (%)	2015 n (%)	*P*
Paracetamol*	295 (98.3; 96.1–99.3)	546 (97.1; 95.4–98.4)	0.656
Ibuprofen*	4 (1.3; 0.5–3.4)	12 (2.1;1.2–3.7)	0.40
Other	1 (0.3; 0.0–1.8)	4 (0.7; 0.3–1.8)	0.48
Second choice drug			
Paracetamol*	19 (6.3; 4.1–9.7;)	61 (10.8; 8.5–13.7)	0.03
Ibuprofen*	276 (92.0; 88.4–94.6)	495 (88.1; 85.1–90-5)	0.04
Acetylsalicilic acid	2 (0.7; 0.2–2.4)	0 (0.0; 0.0–0.0)	0.12
Other	3 (1.0;0.3–2.9)	6 (1.1; 0.5–2.3)	0.92
Choice of administration of paracetamol			
Oral	249 (83.0; 78.3–86-8)	517 (92.0; 89.4–94.0)	< 0.0001
Rectal	51 (17.0;13.2–21.7)	45 (8.0;6.0–10.5)	< 0.0001
Alternating use			
Yes	34 (11.3; 8.2–15.4)	69 (12.3; 9.8–15.2)	0.40
Use of physical methods			
Together with antipyretic drug	29 (9.7; 6.8–13.5)	36 (6.4; 4.7–8.7)	0.08
Before the antipyretic drug	9 (3.0; 1.6–5.6)	14 (2.3; 14.5–41.4)	0.66
If fever persists	153 (51.0; 45.4–56.6)	242 (43.1; 39.0–47.2)	0.15
Never*	109 (36.3; 31.3–41.9)	270 (48.0; 43.9–52.2)	< 0.0001

Note:* right answer according to the Guidelines of the Italian Paediatric Society recommendation

The use of antipyretics according to the presence of discomfort, and not for a specific cut-off of body temperature, was recommended only by 38.2% of paediatricians (vs. 45.3% in 2012).

Paracetamol was confirmed as the first choice antipyretic drug for 97% of paediatricians (Table 2).

None of the participants recommended acetylsalicylic acid but, unfortunately, a small proportion of paediatricians recommended other drugs, besides paracetamol and ibuprofen (including steroids and metamizole) with an antipyretic purpose (Table 2).

Rectal administration only in case of vomiting was correctly recommended by 86.8% of paediatricians in 2015 vs. 74.7% in 2012 (*P* < 0.0001).

**Fig. 1:**
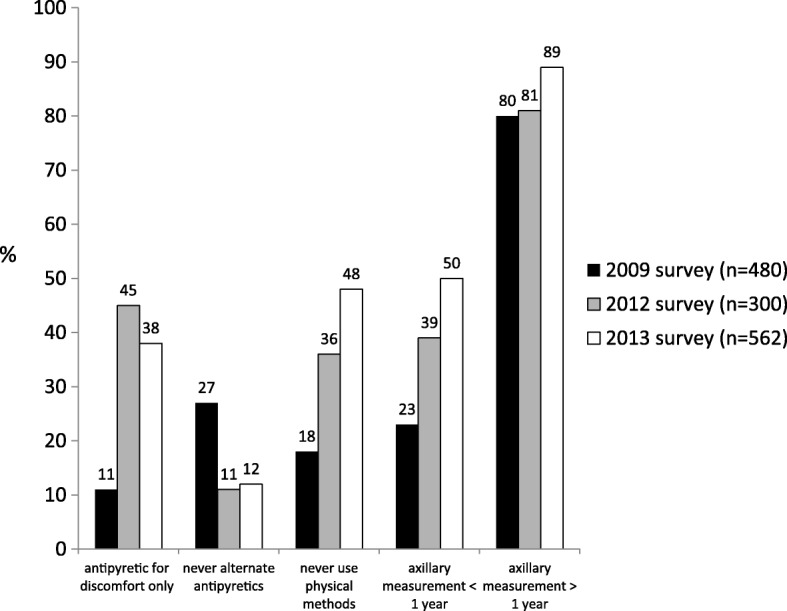
Change in Italian paediatricians’ knowledge over time

The alternate use of paracetamol and ibuprofen was recommended by 12.2% of paediatricians in 2015, similarly to 2012 (Table 2; Fig. 1).

## Discussion

In the present study, changes in knowledge and misconceptions among paediatricians over time were evaluated. Considering also results from our 2009 survey, which included 480 Italian paediatricians [7], progressive improvements in the amount of correct answers were observed (Fig. 1). However, several incorrect habits, including use of physical methods, use of antipyretics not focused on the presence of discomfort, alternate use of antipyretics, and rectal misuse of these drugs are still common.

Several previous studies investigated misconceptions of paediatricians concerning the management of fever in children. Italian pediatricians’ knowledge seems to be in line, or slightly better, than those reported in other European countries in recent years. As an example, the use of physical methods was recommended by 77% of paediatricians in Switzerland [8] and 74% of them [9] in France; this proportion is higher than observed in our study (about 50%). Alternate use of antipyretics was recommended by 65% of Swiss paediatricians [8] but only by 11% of our participants.

The reasons behind the persistence of fever phobia over time have been previously explored [2]. The lack of a strict definition of the child’s discomfort is one major obstacle. Moreover, the evaluation of the child’s discomfort is usually subjectively assessed by one caregiver, and it is influenced by his/her fever-phobia level, in a sort of “vicious circle” [2]. Another issue is the quality of educational material provided by the Scientific Societies that has been found to be often unclear [10]. Finally, paediatricians should improve their ability to communicate with caregivers, not only in terms of time spent for the parents’ education, but also in efforts toward an empathetical and trustful connection with the caregiver [11].

Our study has several limitations. Our results may not be generalized to all paediatricians in Italy, since those included represent approximatively 7% of the entire population of the Italian paediatricians [6]. The two surveys were conducted during two different Conferences. Therefore, the two populations of paediatricians may differ in some characteristics. Personal data (i.e. age and residence) of participants were not collected. Hence our results do not provide information regarding possible differences according to the geographical provenience, age, or other variables. Moreover, adjustment for potential confounders was not possible. It is well-known that self-reported behaviors can be misleading, since some participants might not complete the survey as carefully as they would do in real settings [6]. Participants could be more interested in fever management than non-responding paediatricians. On the other hand, we were able to administer the same questionnaire to large samples of Italian pediatricians, from 2009 to 2015, exploring changes in the adherence to the guideline’s recommendations over time and which messages should be strengthened (Table 3).

**Table 3:** Main recommendations by the Italian fever guidelines [4]

✓ Rectal measurement should not be used routinely in children aged < 5 years because it is invasive and causes discomfort (evidence level III; strength of recommendation D).	
✓ Oral measurement of body temperature should be avoided in children (evidence level III; strength of recommendation D).	
✓ Axillary measurement using a digital thermometer is recommended in children aged < 4 weeks (evidence level III; strength of recommendation B).	
✓ In the hospital or ambulatory care setting, axillary measurement using a digital thermometer or tympanic measurement using an infrared thermometer is recommended in children aged ≥4 weeks (evidence level II; strength of recommendation B).	
✓ For measurements taken at home by parents/caregivers, axillary measurement using a digital thermometer is recommended in all children (evidence level II; strength of recommendation B).	
✓ Use of a tympanic infrared thermometer is not recommended, as this mode of measurement is prone to operator-related error.	
✓ Use of antipyretics in children is recommended only when the fever is associated with evident discomfort (eg, prolonged crying, irritability, reduced activity, reduced appetite, disturbed sleep) (evidence level I; strength of recommendation B).	
✓ Use of physical methods to reduce fever is not recommended (evidence level I; strength of recommendation E).	
✓ Paracetamol and ibuprofen are the only antipyretic drugs recommended for use in children (evidence level I; strength of recommendation A).	
✓ Use of acetylsalicylic acid in children is not recommended because of the risk of Reye’s syndrome (evidence level III; strength of recommendation E).	
✓ Because of their poor benefit– risk ratio, steroids should not be used as antipyretics in children (evidence level III; strength of recommendation E).	
✓ Combined or alternating use of ibuprofen and paracetamol is not recommended (evidence level VI; strength of recommendation D).	
✓ Rectal administration of antipyretics should be considered only in the presence of vomiting or other conditions that prevent oral administration (evidence level I; strength of recommendation A).	

## Conclusion

Our study highlights improvements in the management of the febrile child in Italy, but also detected the persistence of some incorrect habits. Several key messages of the IFG must be further stressed. In particular, the recommendation regarding the use of antipyretics according to the child’s discomfort seems to be adopted only by a minority of paediatricians. Similarly, recent literature reports suggest that improvements in educational interventions are needed in many European countries [2]. Our results may be of help for targeting educational interventions and adherence to practices recommended by the IFG. Further studies are needed in order to understand “weak points” of the communication between Scientific Societies and pediatricians, as well as between paediatricians and caregivers.

## Appendix

### Questionnaire

1. Where should body temperature be measured in children under one year?

- the armpit
- the rectum
- groin crease
- the mouth
- the ear
- on the forehead

2. Where should the body temperature be measured in children over one year?

- the armpit
- the rectum
- groin crease
- the mouth
- the ear
- on the forehead

3. What kind of thermometer do you suggest to measure temperature?

- mercury-in-glass
- electronic
- auricular
- skin infrared
- plastic strip placed on forehead
- “dummy”
- I don’t suggest any particular thermometer

4. Above what temperature do you administer antipyretics?

- < 37 °C
- 37.5 °C
- 38 °C
- 38.5 °C
- 39 °C
- not exist a temperature cut off, depend on patient malaise

5. Which antipyretic drugs do you usually suggest to use?

- acetaminophen
- ibuprofen
- aspirin
- other (metamizole, betamethasone)

6. When the temperature is not going down quickly, do you believe it is useful to associate two or more antipyretic drugs?

- yes
- no

7. Do you suggest to use physical methods as sponging or ice pack to reduce a child’s body temperature?

- yes, with the antipyretic drug
- yes, before the antipyretic drug
- only if the temperature is not going down after the antipyretic drug
- no, never

8. When do you suggest to administer antipyretic drug rectally?

- it’s more useful
- it’s more practical
- because parents prefer this way
- only in the presence of vomiting

## Abbreviation

IFG: Italian Fever Guideline
